# m6A Modification of ATOX1 Inhibits Acute Myeloid Leukemia Progression by Promoting Cuproptosis

**DOI:** 10.1158/2767-9764.CRC-25-0436

**Published:** 2026-04-01

**Authors:** Jiapei Peng, Xiao Fu, Shujun Li, Changmei Hu, Xielan Zhao, Jie Peng

**Affiliations:** 1 School of Medicine, Jishou University, Jishou, China.; 2 Department of Hematology, Xiangya Hospital Central South University, Changsha, China.; 3 Department of Gastroenterology, Second Xiangya Hospital of Central South University, Changsha, China.

## Abstract

Cuproptosis, a regulated cell death caused by copper-dependent enzyme overactivation in the tricarboxylic acid cycle, leads to proteotoxic stress. Although the copper chaperone human antioxidant protein 1 (ATOX1) plays a key role in cuproptosis, its link to acute myeloid leukemia (AML) progression remains unclear. In this study, elesclomol (ES) or disulfiram (DSF)/Cu was used to induce cuproptosis, and bathocuproine disulfonic acid (BCS) was used to inhibit it. An AML xenograft mouse model was also established to validate their effects in tumor tissue. Our study demonstrated that ATOX1 is downregulated in AML. Knockdown of ATOX1 promoted cell viability and proliferation, reduced the proportion of cells in the G_2_–M phase, and decreased cell death. In contrast, overexpression of ATOX1 produced the opposite outcomes. Moreover, ATOX1 knockdown attenuated ES-/Cu-induced cuproptosis in AML cells, whereas ATOX1 overexpression enhanced it. This promoting effect of ATOX1 overexpression was effectively counteracted by the copper chelator BCS. Delving deeper, we discovered that ATOX1 is subject to N6-methyladenosine (m6A) modification mediated by AlkB homolog 5 (ALKBH5). Consequently, ALKBH5 can influence cuproptosis in AML cells by regulating ATOX1 expression. *In vivo*, the role of the ALKBH5–ATOX1 axis in AML progression has also been confirmed. In conclusion, the demethylase ALKBH5 downregulates ATOX1 by reducing its m6A levels, thereby modulating cuproptosis in AML—a mechanism that offers potential novel insights and therapeutic targets for AML treatment.

**Significance:**

This study reveals that in AML, the demethylase ALKBH5 downregulates ATOX1 expression by reducing its m6A modification, thereby inhibiting cuproptosis and promoting AML progression. This mechanism provides a novel potential therapeutic target.

## Introduction

Acute myeloid leukemia (AML), the predominant form of leukemia in adults, is a highly aggressive blood-related cancer. It is marked by the aberrant, unrestrained growth of immature cells within the myeloid lineage. These abnormal cells rapidly accumulate in the bone marrow, effectively crowding it out and severely disrupting the normal process of blood cell production (1, 2). This disease arises from mutations and chromosomal translocations that target various factors, including epigenetic regulators (3). Although intensive chemotherapy can improve the prognosis of patients with AML, the majority of patients ultimately succumb to the disease, with fewer than 30% of patients with AML surviving beyond 5 years and experiencing severe toxicities such as anemia, bleeding, and infections due to the side effects of treatment (4). Hence, there is a pressing demand for the development of more potent and precisely tailored therapeutic approaches for managing human AML.

Copper plays a fundamental role as a trace element in biological systems and is essential for supporting enzyme activity, transcription factor function, and overall cellular health (5). Nonetheless, the accumulation of excess copper within cells can trigger oxidative stress and interfere with cellular processes, underscoring the importance of maintaining precise control over copper levels to ensure proper cellular function. A recent study identified an alternative type of cell death triggered by copper, known as cuproptosis, which operates through a unique mechanism distinct from other cell death pathways (6). Cuproptosis involves copper interacting with lipoic acid enzymes in the tricarboxylic acid cycle, causing protein aggregation, proteotoxic stress, and eventual cell demise (7). Research has linked cuproptosis to the prognosis of various cancers, with the regulatory factor  ferredoxin-1 (FDX1) being notably downregulated in hepatocellular carcinoma. Higher levels of FDX1 expression have been associated with extended survival times in these cases (8). Furthermore, research indicates that serum copper levels may function as prognostic indicators for individuals with AML (9), and long noncoding RNAs linked to cuproptosis are correlated with AML prognosis (10). Despite these findings, the specific contribution of cuproptosis to AML development remains unclear. Therefore, understanding the mechanism of action in AML holds promise for providing novel therapeutic approaches.

The human antioxidant protein 1 (ATOX1) is a copper molecular chaperone that significantly influences cellular antioxidant defense and copper homeostasis (11). ATOX1, a protein located in the cytoplasm, plays a key role in shuttling copper ions within cells to support the function of various copper-dependent enzymes, including those crucial for protecting against oxidative stress and promoting angiogenesis (12). Disruption of the ATOX1-mediated copper balance can lead to elevated oxidative stress and changes in cellular behavior, potentially contributing to the initiation and progression of cancer (13). Research has shown that patients with AML with high levels of ATOX1 expression tend to have notably better survival outcomes than those with low ATOX1 levels (14). However, the regulatory role and underlying mechanisms in AML remain unknown.

In this study, we employed bioinformatics methods to analyze the expression of ATOX1 in The Cancer Genome Atlas (TCGA), Genotype-Tissue Expression (GTEx), and Gene Expression Omnibus (GEO) databases, confirming that ATOX1 is underexpressed in AML. Subsequently, we explored the regulatory role of ATOX1 in cuproptosis in AML pathogenesis through *in vitro* experiments. For further mechanistic investigations, we utilized the RM2Target database to predict the key molecules that may mediate the N6-methyladenosine (m6A) modification of ATOX1. By combining bioinformatics analysis with *in vivo* and *in vitro* validation experiments, we found that the demethylase AlkB homolog 5 (ALKBH5) can inhibit ATOX1 expression by reducing its m6A levels, thereby participating in the regulation of cuproptosis in AML cells. Through these studies, we aimed to provide novel insights into targeted therapeutic strategies for AML.

## Materials and Methods

### Bioinformatics analysis

We obtained bone marrow samples from patients with AML and normal bone marrow samples from TCGA (https://www.genome.gov/Funded-Programs-Projects/Cancer-Genome-Atlas), GTEx (https://gtexportal.org/), and GEO (http://www.ncbi.nlm.nih.gov/geo) databases, all of which are publicly accessible repositories. Publicly available gene expression datasets were analyzed to investigate ATOX1 expression patterns. For the chronic myeloid leukemia (CML) versus normal comparison, data from the GEO series GSE33075 were used. Normal samples included GSM818813 to GSM818819, GSM818824, and GSM818825 (*n* = 9); CML samples comprised GSM817258, GSM817304, and GSM818670 to GSM818685 (*n* = 18). Raw data were normalized using quantile normalization. As all samples belonged to the same batch, no batch correction was applied. Differential expression of ATOX1 between CML and normal samples was assessed using a two-sample *t* test. All analysis scripts are publicly available in the GitHub repository at https://github.com/cmutd/TCGA_GTEx and https://github.com/6YuchengY/GEO-. Gene expression analysis was performed using the R packages “limma” and “DESeq2.” Additionally, the m6A methylation modification status of ATOX1 was predicted using the RMBase v3.0 database (https://rna.sysu.edu.cn/rmbase3/) and the RM2Target database (http://rm2target.canceromics.org).

### Cell culture and treatment

Normal bone marrow mononuclear cells (NBMMC; CP-H238, Procell) and AML cells [HL-60 (RRID: CVCL_0002, iCell-h098, iCell), THP-1 (RRID: CVCL_0006, AW-CCH098, Abiowell), KG-1 (RRID: CVCL_0374, AW-CCH120, Abiowell), and NOMO-1 (RRID: CVCL_1609, iCell-h524, iCell)] were cultivated in growth medium formulations supplemented with FBS and antibiotics. All cell lines underwent rigorous validation via surface plasmon resonance spectroscopy and were subjected to quarterly *Mycoplasma* contamination screening using validated PCR-based detection kits. Cells were maintained in a controlled environment at 37°C and 5% CO_2_. The cells were treated with different concentrations (0, 2, 10, 50, and 200 nmol/L) of the copper ionophore elesclomol (ES) or disulfiram (DSF)/Cu [ES (HY-12040, MedChemExpress) or DSF (HY-B0240, MedChemExpress) and CuCl_2_ (451665-5G, Sigma) combined in a 1:1 ratio] for 72 hours (15). To verify the intrinsic mechanism of ATOX1 in cuproptosis, cells were treated with the copper chelator bathocuproine disulfonic acid (BCS; 1 mmol/L, B1125, Sigma) and ES/Cu (200 nmol/L) for 72 hours (16).

### Cell transfection

Cell transfection was performed using Lipofectamine 2000 (11668019, Invitrogen). The following plasmids were used for cell transfection: sh-*ATOX1* (HG-SH004045), sh-*FDX1* (HG-SH004109), sh-*ALKBH5* (HG-SH017758), oe-*ATOX1* (HG-HO121022), and oe-*ALKBH5* (HG-HO17758), and their blank control plasmids sh-NC and oe-NC. The above plasmids were purchased from HonorGene. The sequences of short hairpin RNAs (shRNA) were as follows: sh-*ATOX1* #1, 5′-GCTGTGCTGAAGCTGTCTCTC-3′; sh-*ATOX1* #2, 5′-ACAGACCTG GACTTGGCAGT-3′; sh-*ATOX1* #3, 5′-AGGAGTTAAGTATGACATTGA-3′; sh-*FDX1*, 5′-GTGGTTGAAAATAATCTAGAT-3′; sh-*ALKBH5*, 5′- GAGCATATGCGTGTGATTATT-3′; and sh-NC, 5′-TTAACGTAGCAGTTAGGAGGC-3′.

### qRT-PCR

After isolating RNA with TRIzol, it was converted into cDNA using an mRNA Reverse Transcription Kit (CW2569, CWBIO). Subsequently, gene expression analysis was performed on the ABI 7900 system utilizing the Ultra SYBR Mixture (CW2601, CWBIO). The housekeeping gene *β-actin* was employed as an internal control for quantifying mRNA levels. The 2^−ΔΔCt^ method was used to determine the relative expression levels, with the primer sequences detailed in Table 1.

**Table 1.** Primer sequences for qRT-PCR.

Genes	Sequences (5′–3′)
*β-* *Actin*_Fwd	ACC​CTG​AAG​TAC​CCC​ATC​GAG
*β-* *Actin*_Rev	AGC​ACA​GCC​TGG​ATA​GCA​AC
*ATOX1*_Fwd	CTG​AAG​CTG​TCT​CTC​GGG​TC
*ATOX1*_Rev	TTC​AGG​GTT​GCA​AGC​AGA​GT

### Western blotting

For protein extraction, both cells and tissue samples were lysed using RIPA buffer (AWB0136, Abiowell). Quantification of the extracted protein was carried out using a bicinchoninic acid assay kit (AWB0104, Abiowell). Subsequently, the total protein was separated via SDS-PAGE and transferred onto nitrocellulose membranes using an electrophoretic transfer technique. The membranes were then incubated with primary antibodies overnight at 4°C. Following this step, they were treated with a horseradish peroxidase–linked secondary antibody solution, and visual detection was facilitated by applying an enhanced chemiluminescence reagent. Normalization was done using β-actin as the internal reference. Details of the primary antibodies can be found in Table 2.

**Table 2.** Primary antibodies for Western blotting.

Name	Code	Dilution ratio	Source	RRID
ATOX1	22641-1-AP	1:2,000	Proteintech	AB_2879139
FDX1	12592-1-AP	1:2,000	Proteintech	AB_11182486
LIAS	AWA11095	1:1,000	Abiowell	—
DLAT	AWA10279	1:1,000	Abiowell	—
ACO2	11134-1-AP	1:5,000	Proteintech	AB_2289288
METTL3	15073-1-AP	1:20,000	Proteintech	AB_2142033
ALKBH5	16837-1-AP	1:5,000	Proteintech	AB_2242665
ELAVL1	ab200342	1:1,000	Abcam	AB_2784506
HNRNPC	ab133607	1:20,000	Abcam	AB_2860560
β-Actin	66009-1-Ig	1:5,000	Proteintech	AB_2687938

### Cell Counting Kit-8 assay

Cells were seeded in 96-well plates at a density of 5 × 10^3^ cells per well in 100 μL of culture medium. After incubation, 10 μL of Cell Counting Kit-8 reagent (NU679, Dojindo) was added to each well, and the plates were incubated in an incubator at 37°C for 4 hours. Cell viability was assessed by measuring the absorbance at 450 nm using a HEALES microplate reader model MB-530.

### EDU staining

An EDU Assay Kit (C10310, RiboBio) was used to analyze cell proliferation. Cells exposed to different treatments were plated in 96-well plates at a density of 5 × 10^4^ cells per well. Subsequently, each well was treated with a diluted EDU solution and allowed to incubate overnight. The wells underwent a series of steps, including fixation with 4% paraformaldehyde, treatment with glycine and Triton X-100, Apollo staining, and Hoechst 33342 staining. Positive labeling was visualized using fluorescence microscopy (CX41-72C02, Olympus) and quantified.

### Detection of cell cycle by flow cytometry

After resuspending the cells in cold PBS, the suspension was centrifuged (800 rpm for 5 minutes). The supernatant was discarded, 400 μL of PBS was added, and the cells were gently dispersed. The cells were then gradually mixed in 1.2 mL of absolute ethanol dropwise under ice-bath conditions (to achieve a final concentration of 75%), and the cells were fixed overnight at 4°C. The following day, the cells were collected and washed twice with cold PBS to remove residual ethanol. The cells were incubated with 150 μL of propidium iodide (PI) staining solution in the dark at 4°C for 30 minutes. For flow cytometry detection, a 488-nm excitation light/630-nm emission filter system was used to acquire signals from 10,000 single cells (excluding debris and cell aggregates by double-parameter gating with forward scatter and side scatter). The proportions of cells in the G_0_–G_1_, S, and G_2_–M phases were analyzed using ModFit software.

### Detection of cell death by flow cytometry

Cells were dissociated with trypsin, harvested by spinning at 2,000 rpm for 5 minutes, and resuspended in 500 μL of binding buffer. To the cell suspensions, 5 μL of PI was added, followed by an incubation period of 10 minutes. Flow cytometry was utilized to analyze the stained cell samples.

### RNA pull-down assay

Magnetic beads labeled with streptavidin were incubated with biotin-labeled RNA. Cells were lysed using a cell lysate buffer to which protease inhibitors were added in advance. Subsequently, the RNA-bound magnetic beads were incubated with the cell lysate, allowing the capture of all molecules capable of binding to RNA. These bound molecules were then eluted, and the proteins in the eluate were further analyzed by Western blotting.

### RNA immunoprecipitation-qPCR

RNA immunoprecipitation (RIP) assays were conducted by using the RIP kit (17–700) manufactured by Millipore Corporation, following the manufacturer’s instructions. Briefly, cells were lysed with a lysis buffer and incubated with an m6A monoclonal antibody (68055-1-Ig, Proteintech, RRID: AB_2918796) or an IgG control group antibody. After immunoprecipitation, qRT-PCR analysis was performed to detect the enrichment of *ATOX1* RNA. The primer sequences were as follows: *ATOX1*-forward, 5′-CTG​AAGCTG​TCT​CTC​GGG​TC-3′; *ATOX1*-reverse, 5′-TTC​AGG​GTT​GCA​AGC​AGA​GT-3′.

### RNA stability assay

Cells were cultured in six-well plates and exposed to actinomycin D (ActD) at a concentration of 5 μg/mL (Sigma-Aldrich) for different durations (2, 4, and 8 hours). The negative control group, which was treated with DMSO, was sampled at the start (0 hours). Subsequently, the total RNA was isolated using the phenol–chloroform–ethanol method and subjected to qPCR analysis. The mRNA decay rate was determined by calculating the half-life using linear regression analysis.

### Animals and treatment

The animal experiments were reviewed and approved by the Experimental Animal Ethics Committee of Xiangya Hospital of Central South University (no. 2023111605). Female BALB/c nude mice (4 weeks old), ordered from the Hunan Slake Jinda Laboratory Animal Center, were subcutaneously injected with NOMO-1 cells in the right axilla 1 week after acclimatization. Into each BALB/c mouse, 1 × 10^7^ cells were injected. The injection volume was 100 μL. After tumor implantation, tumor measurements and observations were performed twice a week. Mice with tumors were equally divided into four groups: oe-NC + oe-NC, oe-*ATOX1* + oe-NC, oe-*ALKBH5* + oe-NC, and oe-*ALKBH5* + oe-*ATOX1*. Mice in each group were injected intratumorally with the corresponding lentivirus (2 × 10^9^ TU) on days 7, 12, and 17 after tumor seeding. The experiment was completed over 21 days. The tumor-bearing rats were photographed and collected.

### IHC

The tissue sections were dewaxed using xylene and rehydrated using an ethanol series. They were then exposed to a 50% uric acid solution at 37°C for 30 minutes and subsequently treated with trypsin solution at the same temperature for another 30 minutes. Next, the sections were treated with 1% periodate for 10 minutes to deactivate the endogenous enzymes. The slices were incubated with the primary antibody Ki67 (AWA10320, Abiowell) overnight at 4°C, followed by incubation with anti–rabbit IgG H&L at 37°C for 30 minutes. DAB (ZLI-9018, Beijing Zhongsui Jinqiao Biotechnology Co.) and hematoxylin (AWI0001a, Abiowell) were used for color development and nuclear staining, respectively.

### Statistical analysis

Each experiment was conducted a minimum of three times to ensure reproducibility. Quantitative data are presented as mean ± SD. Statistical evaluations were performed using GraphPad Prism 8.0 software, employing two-tailed Student *t* test for pairwise comparisons, one-way ANOVA for multi-group analysis, or Kruskal–Wallis H test for nonparametric data. For *post hoc* assessments following one-way ANOVA, the Tukey test was applied to identify significant differences between specific groups. When the Kruskal–Wallis test indicated statistical significance, the Dunn method was utilized for pairwise comparisons to determine precise group differences.

## Results

### ATOX1 is lowly expressed in AML

**Figure 1.**
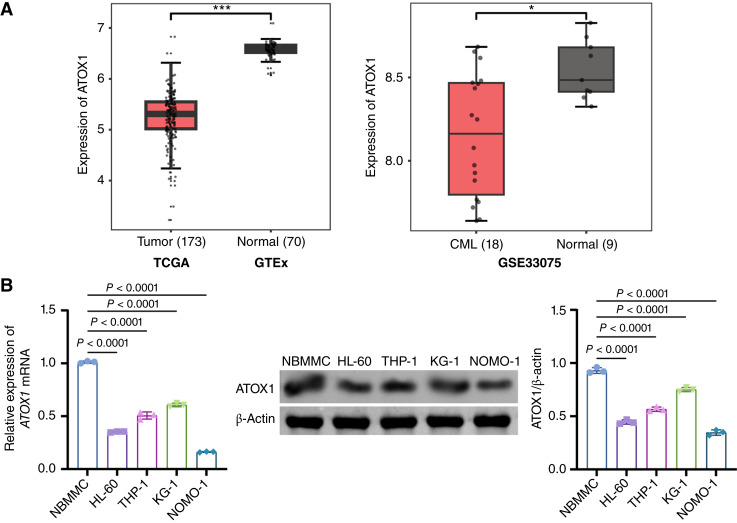
ATOX1 is lowly expressed in AML. **A,** Bioinformatics analysis of ATOX1 expression in bone marrow of normal individuals and patients with AML (data obtained from TCGA, GTEx, and GEO databases, respectively). ***, *P* < 0.001; *, *P* < 0.05. **B,** qRT-PCR and Western blot analysis of ATOX1 expression in NBMMCs, HL-60, THP-1, KG-1, and NOMO-1 cells. Data are shown as mean ± SD. *n* = 3.

In light of the potential pivotal role of ATOX1 in the progression of AML, we employed bioinformatics approaches to examine the expression levels of ATOX1 in the TCGA database and the GSE33075 and GSE1159 datasets from the GEO database. The analyses consistently revealed that ATOX1 levels were lower in the bone marrow of patients with AML than those in healthy individuals (Fig. 1A). Furthermore, we conducted assays using NBMMCs and AML cell lines (HL-60, THP-1, KG-1, and NOMO-1). Consistent with the bioinformatics findings, ATOX1 expression was lower in AML cell lines than in NBMMCs (Fig. 1B), with the lowest expression observed in HL-60 and NOMO-1 cells. Collectively, these observations indicate that ATOX1 is underexpressed in AML.

### ATOX1 overexpression alleviates AML progression

**Figure 2.**
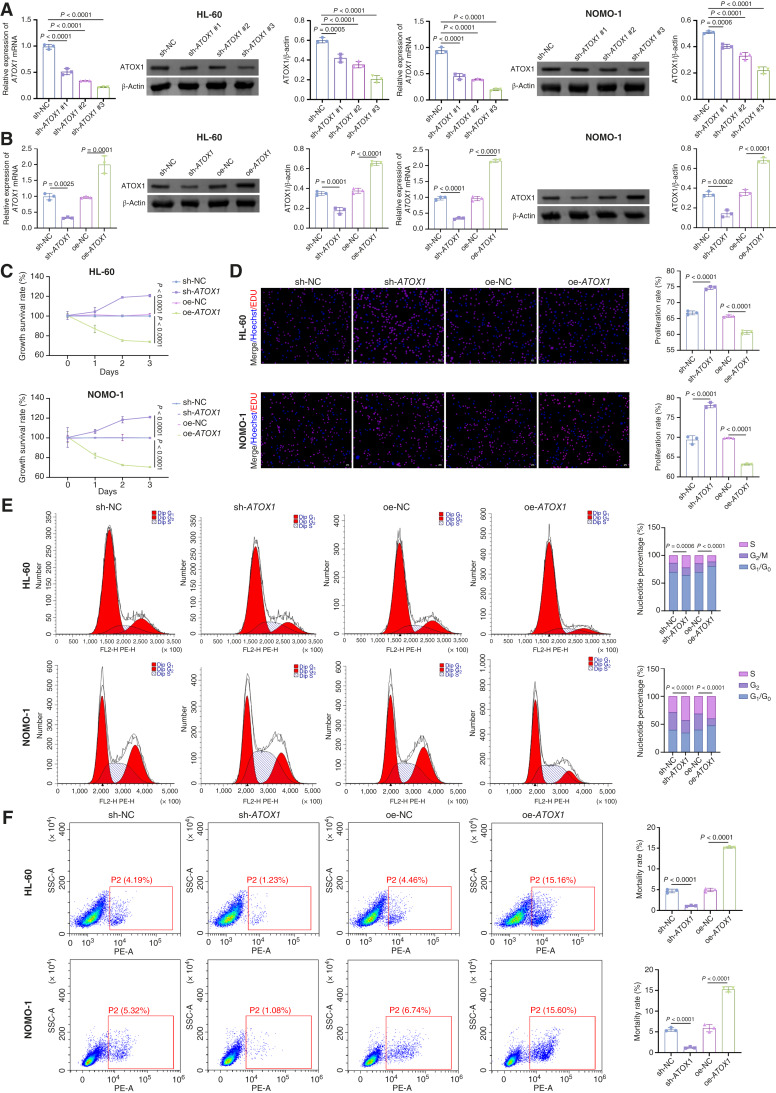
ATOX1 overexpression alleviates AML progression. **A,** qRT-PCR and Western blot analysis of ATOX1 expression in AML cells transfected with sh-NC or sh-*ATOX1*. AML cells were transfected with sh-*ATOX1* or oe-A*TOX1*. **B,** Western blot analysis of ATOX1 expression in AML cells. **C,** Cell Counting Kit-8 assay for detecting the viability of AML cells. **D,** EDU staining assay for detecting the cell proliferation of AML cells. Scale bar, 25 μm. **E,** Flow cytometry for detecting the cell cycle of AML cells. **F,** Flow cytometry for detecting the cell death of AML cells. Mortality rate (%) represents the percentage of PI-positive (membrane-compromised necrotic/late apoptotic) cells. Data are shown as mean ± SD. *n* = 3. SSC, side scatter.

Subsequently, we generated ATOX1 knockdown and overexpression AML cell lines to elucidate the role of ATOX1 in AML progression. To ensure the efficacy of the knockdown, we initially conducted an assessment. We observed that sh-*ATOX1* #3 exhibited the most effective knockdown (Fig. 2A). As depicted in Fig. 2B, sh-*ATOX1* significantly reduced ATOX1 expression, whereas oe-*ATOX1* markedly increased it. In our evaluation of cellular functions, we found that ATOX1 knockdown led to increased viability and proliferative capacity of AML cells. This was accompanied by reduced G_2_–M-phase accumulation and a decrease in cell death (Fig. 2C–F). Conversely, ATOX1 overexpression led to reduced proliferation of AML cells, along with increased G_2_–M-phase accumulation and a higher level of cell death (Fig. 2C–F). During the 7-day observation period, sh-*ATOX1* consistently demonstrated a robust ability to promote cell proliferation and inhibit cell death, whereas oe-*ATOX1* exhibited opposing effects (Supplementary Fig. S1A and S1B). To perform a rescue experiment, we expressed a shRNA-resistant (silent-mutated) *ATOX1* cDNA in the sh-ATOX1–treated AML cells. This restored ATOX1 expression (Supplementary Fig. S2A) and, importantly, reversed the functional consequences of ATOX1 knockdown: the rescue offsets the enhancement of cell viability and proliferation (Supplementary Fig. S2B–S2D) and the suppression of cell death (Supplementary Fig. S2E) induced by sh-*ATOX1*. Together, these results demonstrate that re-expression of ATOX1 rescues the functional defects caused by its knockdown, confirming the specificity of the observed phenotypes. These findings suggest that ATOX1 overexpression effectively mitigates AML progression.

### ATOX1 overexpression promotes DSF-/Cu-induced cuproptosis in AML cells

**Figure 3.**
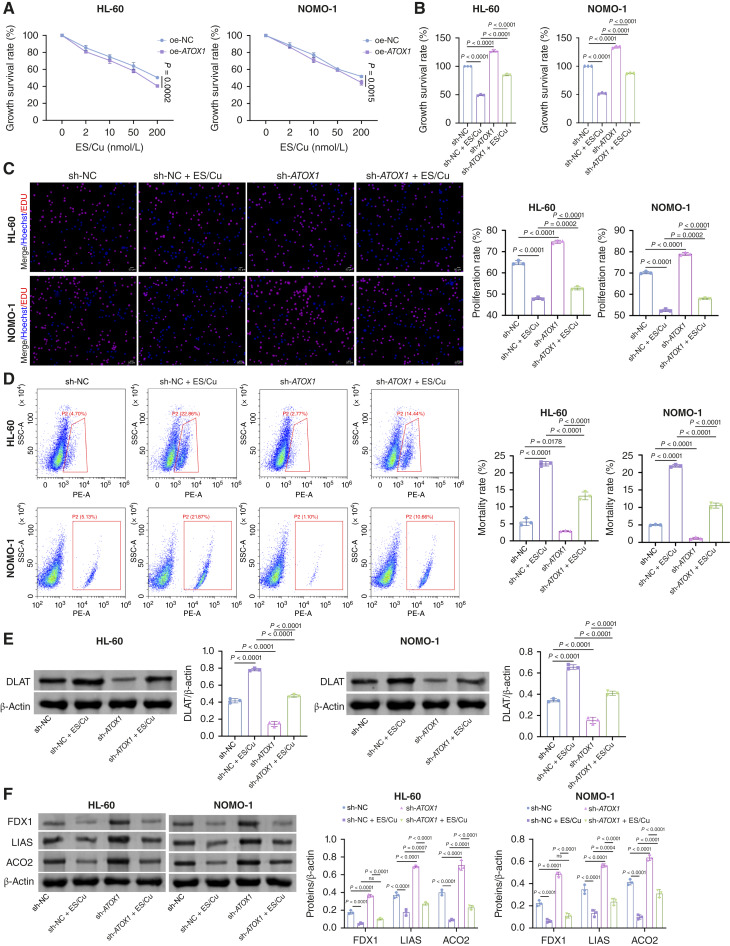
ATOX1 knockdown inhibits ES-/Cu-induced cuproptosis in AML cells. **A,** Cell Counting Kit-8 assay for detecting the viability of AML cells transfected with oe-NC or oe-ATOX1 treated with different concentrations of ES/Cu (0, 2, 10, 50, and 200 nmol/L and ES and CuCl_2_ combined in a 1:1 ratio). AML cells transfected with sh-NC or sh-*ATOX1* were treated with 200 nmol/L ES/Cu for 72 hours. **B,** Cell Counting Kit-8 assay for detecting the viability of AML cells. **C,** EDU staining assay for detecting the cell proliferation of AML cells. Scale bar, 25 μm. **D,** Flow cytometry for detecting the cell death of AML cells. **E,** Western blot analysis of lipoylated DLAT expression in AML cells. **F,** Western blot analysis of Fe–S cluster protein (FDX1, LIAS, and ACO2) expression in AML cells. Data are shown as mean ± SD. *n* = 3. SSC, side scatter.

As ATOX1 serves as a crucial copper chaperone involved in cuproptosis pathways (17), we investigated the impact of ES or DSF combined with copper, at a 1:1 ratio, on the survival of cells overexpressing ATOX1. These findings revealed a gradual reduction in the viability of AML cells with increasing concentrations of ES/Cu or DSF/Cu. Furthermore, upon treatment with oe-*ATOX1*, cell viability decreased even further (Fig. 3A; Supplementary Fig. S3A). Compared with the sh-NC group, the sh-NC + ES/Cu group exhibited reduced cell viability and proliferation rate alongside increased mortality rates. Notably, knockdown of ATOX1 significantly reversed these trends (Fig. 3B–D). The hallmark of cuproptosis is aggregation of lipoylated dihydrolipoamide S-acetyltransferase (DLAT) and loss of iron–sulfur (Fe–S) proteins (FDX1, LIAS, and ACO2; ref. 18). ES/Cu treatment enhanced the levels of lipoylated DLAT while decreasing the expression of Fe–S cluster proteins (FDX1, LIAS, and ACO2). In contrast, ATOX1 knockdown decreased lipoylated DLAT levels and increased the expression of these Fe–S cluster proteins (Fig. 3E and F). Collectively, these findings indicate that ATOX1 knockdown suppresses ES-/Cu-induced cuproptosis.

**Figure 4.**
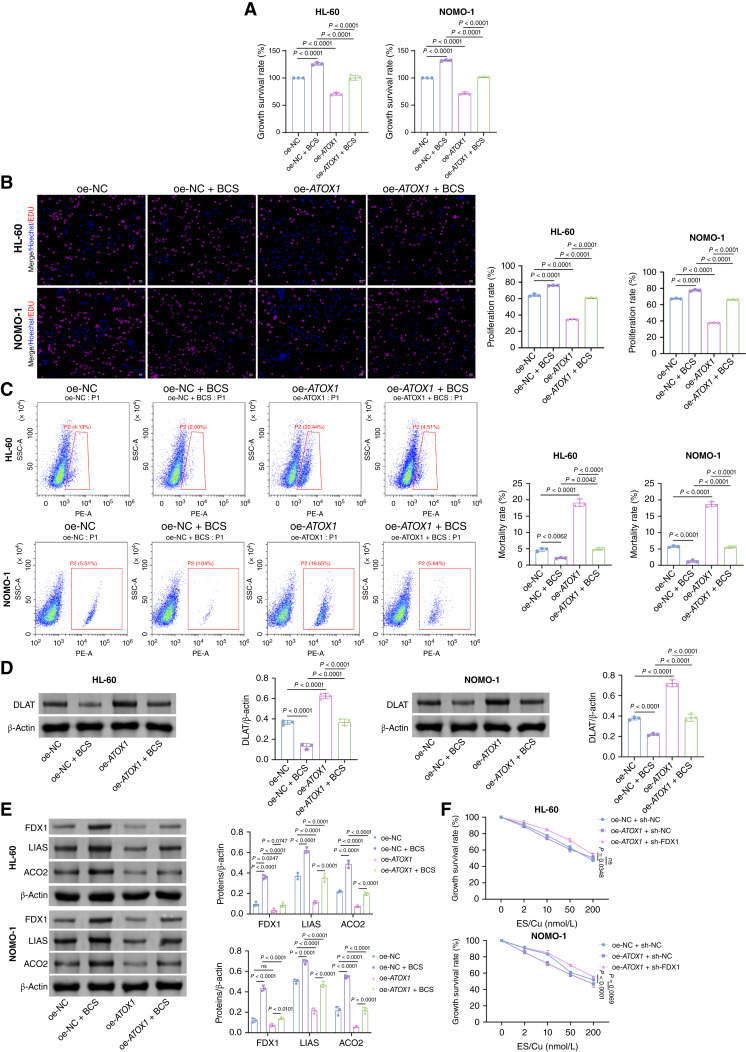
ATOX1 overexpression promotes ES-/Cu-induced cuproptosis in AML cells. AML cells transfected with oe-NC or oe-*ATOX1* were treated with 200 nmol/L ES/Cu for 72 hours. **A,** Cell Counting Kit-8 assay for detecting the viability of AML cells. **B,** EDU staining assay for detecting the cell proliferation of AML cells. Scale bar, 25 μm. **C,** Flow cytometry for detecting the cell death of AML cells. **D,** Western blot analysis of lipoylated DLAT expression in AML cells. **E,** Western blot analysis of Fe–S cluster protein (FDX1, LIAS, and ACO2) expression in AML cells. **F,** Cell Counting Kit-8 assay for detecting the viability of AML cells transfected with oe-NC or oe-ATOX1 and sh-NC or sh-*FDX1* treated with different concentrations of ES/Cu (0, 2, 10, 50, and 200 nmol/L) for 72 hours. Data are shown as mean ± SD. *n* = 3. SSC, side scatter.

Additionally, we treated the cells with the cuproptosis inhibitor BCS. Compared with the oe-NC group, the oe-NC + BCS group exhibited significantly enhanced cell viability and proliferation rate, with reduced G_2_–M-phase accumulation and cell death (Fig. 4A–C). The oe-*ATOX1* group showed an opposite trend. Similarly, BCS exhibited similar effects in ATOX1-overexpressing cell lines. As illustrated in Fig. 4D and E, BCS treatment diminished lipoylated DLAT levels and augmented Fe–S cluster protein expression (FDX1, LIAS, and ACO2). Conversely, overexpression of ATOX1 reversed these effects, restoring lipoylated DLAT while reducing Fe–S cluster protein levels. Moreover, the cuproptosis induced by ATOX1 overexpression was suppressed by knockdown of FDX1, a key regulator of the cuproptosis pathway (Fig. 4F). These findings suggest that ATOX1 overexpression promotes ES-/Cu-induced cuproptosis in AML cells.

### ATOX1 expression is affected by ALKBH5-mediated m6A modification

**Figure 5.**
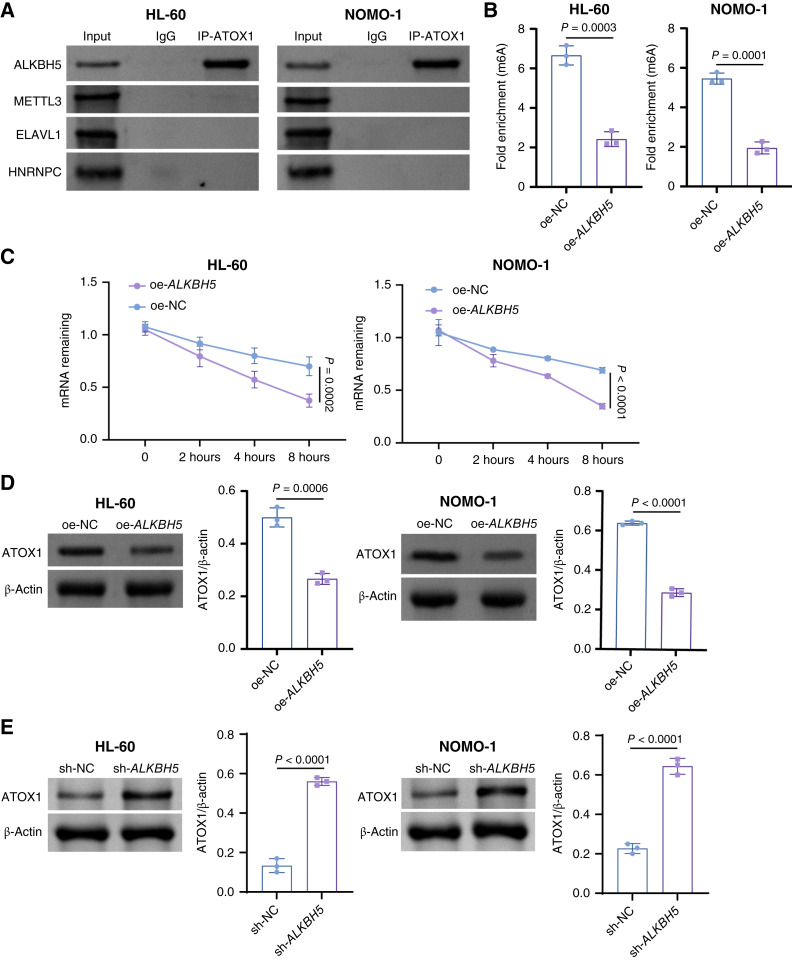
ATOX1 expression is affected by ALKBH5-mediated modification of m6A methylation. **A,** RNA pull-down assay for identifying the interaction of ATOX1 RNA with METTL3, ALKBH5, ELAVL1, and HNRNPC. AML cells were transfected with oe-NC or oe-*ALKBH5*. **B,** RIP-qPCR assay for detecting the m6A methylation level of *ATOX1* in AML cells. **C,** qRT-PCR of *ATOX1* transcripts in ActD-treated AML cells. **D,** Western blot analysis of ATOX1 expression in AML cells. AML cells were transfected with sh-NC or sh-*ALKBH5*. **E,** Western blot analysis of ATOX1 expression in AML cells. Data are shown as mean ± SD. *n* = 3.

Growing evidence has emphasized the importance of disrupted epigenetic mechanisms in driving the development of AML (2). Among these mechanisms, m6A is the most prevalent RNA modification in mammalian mRNA (19). In AML, various m6A-related proteins exhibit abnormal expression patterns and have been implicated in both promoting and suppressing tumor growth, thereby influencing disease onset and advancement (20). Utilizing the RMBase v3.0 database, we predicted multiple m6A modification sites on ATOX1. However, reports on m6A methylation modification of *ATOX1* are scarce. To address this, we employed the RNA modification database RM2Target to predict that *ATOX1* RNA might be modified by m6A writer protein METTL3, m6A eraser protein ALKBH5, and m6A reader proteins ELAVL1 and HNRNPC in human leukemia cell lines. To validate these predictions, we conducted RNA pull-down experiments to investigate the interactions between *ATOX1* RNA and several proteins, including METTL3, ALKBH5, ELAVL1, and HNRNPC. Our findings revealed a specific interaction between *ATOX1* RNA and ALKBH5 (Fig. 5A). Additionally, we established AML cell lines overexpressing ALKBH5 to confirm its regulatory impact on ATOX1. Remarkably, overexpression of ALKBH5 led to a reduction in the m6A level of *ATOX1* RNA (Fig. 5B). Concurrently, we observed the degradation rate of *ATOX1* mRNA in ActD experiments, which demonstrated that ALKBH5 overexpression accelerated *ATOX1* mRNA degradation (Fig. 5C). Western blot results also indicated that ALKBH5 overexpression decreased ATOX1 expression and ALKBH5 knockdown decreased ATOX1 expression (Fig. 5D and E). Collectively, these findings suggest that ALKBH5 promotes *ATOX1* degradation and reduces its expression by lowering m6A levels.

### ALKBH5-mediated m6A modification regulating ATOX1 expression affects cuproptosis in AML cells

**Figure 6.**
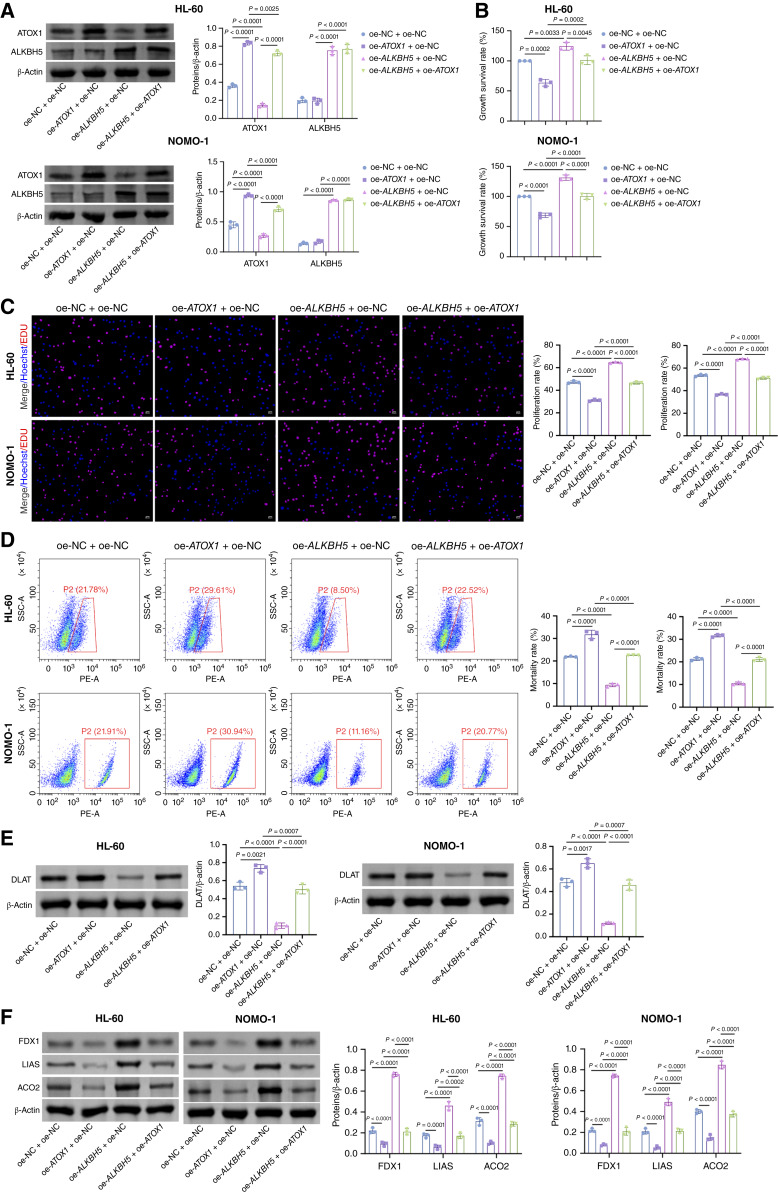
ALKBH5-mediated m6A modification regulating ATOX1 expression affects cuproptosis in AML cells. AML cells transfected with oe-*ATOX1* and/or oe-*ALKBH5* were treated with 200 nmol/L ES/Cu for 72 hours. **A,** Western blot analysis of ATOX1 and ALKBH5 expression in AML cells. **B,** CCK-8 assay for detecting the viability of AML cells. **C,** EDU staining assay for detecting the cell proliferation of AML cells. Scale bar, 25 μm. **D,** Flow cytometry for detecting the cell death of AML cells. **E,** Western blot analysis of lipoylated DLAT expression in AML cells. **F,** Western blot analysis of Fe–S cluster protein (FDX1, LIAS, and ACO2) expression in AML cells. Data are shown as mean ± SD. *n* = 3. SSC, side scatter.

The regulatory effect of ALKBH5 on the m6A methylation modification of *ATOX1* has been established. Subsequently, we explored the effect of ALKBH5 on ATOX1-mediated cuproptosis in AML cells. To this end, we treated AML cells overexpressing ATOX1, ALKBH5, or both with ES/Cu or DSF/Cu. Western blot results illustrated the protein expression levels of ATOX1 and ALKBH5 in each treatment group. Overexpression of ATOX1 significantly augmented ATOX1 expression without notably affecting ALKBH5 levels. Conversely, ALKBH5 overexpression markedly elevated ALKBH5 expression, while reducing ATOX1 levels (Fig. 6A). Consistent with prior findings, ATOX1 overexpression significantly reduced cell viability, proliferation rates, and Fe–S cluster protein levels in AML cells, while concurrently increasing G_2_–M-phase accumulation, elevating lipoylated DLAT levels, and promoting cell death (Fig. 6B–F; Supplementary Fig. S3B–S3G). Interestingly, ALKBH5 overexpression exhibited the opposite effects, and this effect of oe-*ALKBH5* was reversed by oe-*ATOX1*. Collectively, these results suggest that ALKBH5 regulates ATOX1 expression by mediating its m6A modification, thereby influencing cuproptosis in AML cells.

### Animal experiments validate the therapeutic potential of the ALKBH5–ATOX1 axis for AML

**Figure 7.**
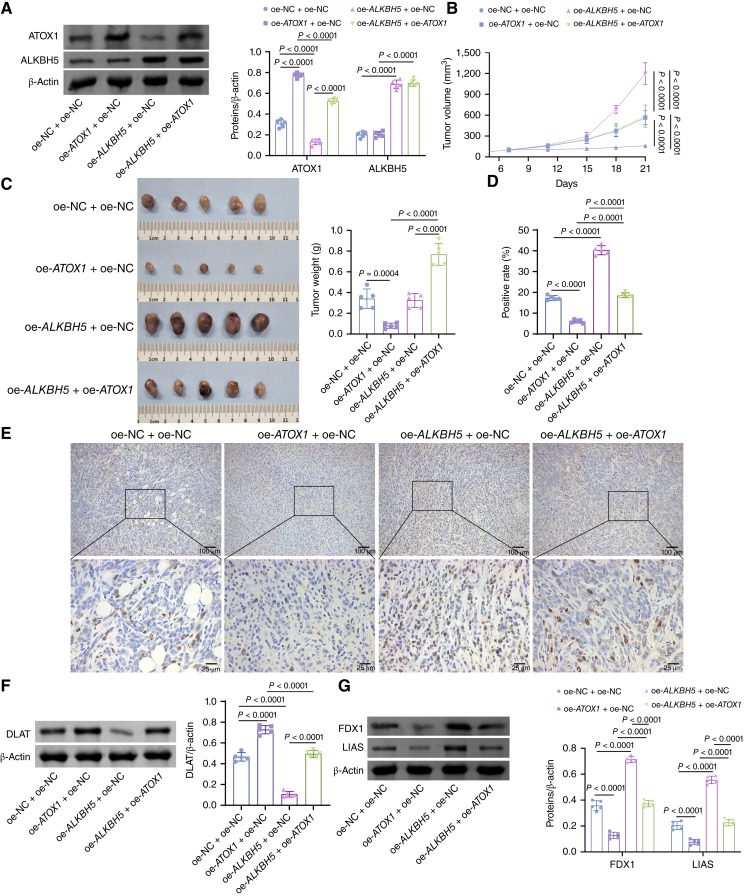
Animal experiments validate the therapeutic potential of the ALKBH5–ATOX1 axis for AML. Female BALB/c nude mice were subcutaneously injected with 1 × 10^7^ NOMO-1 cells in the right axilla. **A,** Western blot analysis of ATOX1 and ALKBH5 expression in tumor tissues of AML model mice. **B,** The growth curve of tumor volume in AML model mice. **C,** Representative images and weight of tumors in AML. **D** and **E,** IHC assay for detecting Ki67 expression. Scale bar, 25 and 100 μm. **F,** Western blot analysis of lipoylated DLAT expression in tumor tissues of AML model mice. **G,** Western blot analysis of Fe–S cluster protein (FDX1 and LIAS) expression in tumor tissues of AML model mice. Data are shown as mean ± SD. *n* = 5.

To comprehensively ascertain the impact of the ALKBH5–ATOX1 axis on AML progression, we constructed an AML xenograft model by subcutaneously injecting NOMO-1 cells into nude mice. Subsequently, we intratumorally injected the viruses overexpressing ATOX1 and/or ALKBH5. Notably, ATOX1 expression was elevated in tumor tissues treated with oe-*ATOX1*, whereas ALKBH5 expression was increased and ATOX1 expression was reduced in tumors treated with oe-*ALKBH5* (Fig. 7A). We observed that ATOX1 overexpression significantly inhibited AML tumor growth, whereas ALKBH5 overexpression promoted it. Interestingly, ATOX1 overexpression reversed the tumor-promoting effects of ALKBH5 overexpression (Fig. 7B and C). IHC results for Ki67 also indicated a decrease in tumor cell proliferation in the oe-*ATOX1* + oe-NC group compared with the oe-NC + oe-NC group and an increase in the oe-*ALKBH5* + oe-NC group. Furthermore, the oe-*ALKBH5* + oe-*ATOX1* group exhibited a higher proliferation rate than the oe-*ATOX1* + oe-NC group and a lower proliferation rate than the oe-*ALKBH5* + oe-NC group (Fig. 6D and E). Additionally, we examined the occurrence of cuproptosis in mouse tumor tissues. IHC analysis of Ki67 revealed a reduced tumor cell proliferation in the oe*-ATOX1* + oe-NC group compared with that in the oe-NC + oe-NC group and an increased proliferation rate in the oe-*ALKBH5* + oe-NC group. Furthermore, the proliferation rate was higher in the oe-*ALKBH5* + oe-*ATOX1* group than in the oe-*ATOX1*+ oe-NC group but lower than that in the oe-*ALKBH5* + oe-NC group (Fig. 7D and E). Complementary investigations have also explored the manifestation of cuproptosis within murine tumor specimens. The results demonstrate that oe-*ATOX1* treatment reduced Fe–S cluster protein levels while elevating lipoylated DLAT levels in AML cells. Conversely, oe-*ALKBH5* treatment induced opposite effects, increasing Fe–S cluster proteins and decreasing lipoylated DLAT. Notably, the molecular changes induced by oe-*ATOX1* were reversed following oe-*ALKBH5* overexpression (Fig. 7F and G). Collectively, these findings suggest that ALKBH5 mediates the regulation of ATOX1 expression, thereby inhibiting cuproptosis and promoting AML progression.

## Discussion

ATOX1 has recently emerged as a key player in tumor advancement, with studies highlighting its role in driving breast cancer cell migration (21). Additionally, investigations have revealed that Activin A promotes migration and colony formation of colon cancer cells by facilitating the translocation of ATOX1 into the nucleus (22). For this research project, we utilized bioinformatics tools to analyze the expression of ATOX1 in AML and confirmed its reduced levels through qRT-PCR and Western blot assays. However, previous research has shown that ATOX1 is highly expressed in AML cells (THP-1) compared with bone marrow stromal cells (23). This discrepancy may stem from variations in protein expression across different cell lines. To elucidate the specific effects of ATOX1 on the biological behavior of AML cells, we conducted a series of cellular experiments. Our findings revealed that ATOX1 knockdown enhanced AML cell viability, promoted cell proliferation, and reduced G_2_–M-phase cell accumulation. Conversely, overexpression of ATOX1 resulted in the opposite effects. Additionally, in AML tumor-bearing mice, we confirmed that ATOX1 overexpression effectively inhibited tumor growth. These results suggest that the overexpression of ATOX1 could serve as an effective approach to alleviate AML. Taken together, these findings indicate that the upregulation of ATOX1 expression may represent a promising approach to combat AML.

Copper metabolism is a complex and tightly regulated process that involves a multitude of molecules operating at both cellular and organ levels. In the cytoplasm, copper ion uptake is facilitated by SLC31A1 and SLC31A2, whereas copper export is managed by the ATPases ATP7A and ATP7B. Within cells, copper is shuttled to different subcellular organelles by various copper-binding proteins, such as COX17, CCS, and ATOX1, to maintain bioavailability (24). Additionally, the utilization of copper ionophores, small molecules capable of transporting copper into cells, plays a significant role in this process, with some showing promise in cancer treatment (25, 26). The two primary ionophores associated with copper reduction are ES and DSF. ES binds to Cu^2+^ in the extracellular environment and transports it into the mitochondrial matrix (27). FDX1 encodes a reductase that reduces Cu^2+^ to its more toxic form Cu^+^, lipoylates DLAT-a process essential for cuproptosis (28)—and serves as the direct target of ES (29). DSF is another known copper ionophore that facilitates copper uptake into cells (7). This ionophore has been explored for its potential in antitumor therapy. Additionally, DSF is recognized as a proteasome target and contributes to the inhibition of proteasome function. The proteasome-mediated degradation pathway is a major focus in the development of many anticancer drugs. When DSF binds with copper to form a complex, it acts as a proteasome inhibitor, targeting copper in tumor cells and leading to cell death (30). Changes in cellular copper levels can stimulate tumor progression, invasion, and drug resistance. Conversely, excess intracellular copper can trigger cuproptosis, a unique form of cell demise (7). Because of the crucial involvement of copper and its influence on cell death mechanisms in cancer development, treatments targeting copper show promise in hindering tumor proliferation, particularly in addressing chemoresistant tumors. This suggests a novel avenue for cancer therapy utilizing copper-based interventions (31). The significance of ATOX1 as a copper chaperone in copper metabolism cannot be overstated. ATOX1 binds to Cu (I) and transports it to ATP7A and ATP7B, located in the Golgi network, for further processing (32). Additionally, ATOX1 can facilitate the transport of copper into the nucleus, in which it acts as a copper-dependent transcription factor (33, 34). In the present study, we induced cuproptosis in AML cells using ES/Cu while employing the cuproptosis chelator BCS to suppress cuproptosis. Notably, ES/Cu treatment significantly inhibited the viability and proliferative capacity of AML cells, increased the accumulation of cells in the G_2_–M phase and the protein levels of Fe–S cluster proteins (FDX1, LIAS, and ACO2), and reduced lipoylated DLAT levels, which are key factors in cuproptosis. These findings indicate that cuproptosis can effectively damage the biological activity of AML cells, consistent with the effects observed in other tumors (35, 36). Furthermore, we discovered that knockdown of ATOX1 significantly inhibited ES-/Cu-induced cuproptosis, whereas overexpression of ATOX1 enhanced this process. Notably, the procuproptotic effect of ATOX1 overexpression was effectively counteracted by BCS treatment and knockdown of FDX1. These research outcomes hold substantial importance for guiding future investigations aimed at leveraging cuproptosis for the precise treatment of AML.

Mounting evidence underscores the significant role of perturbed epigenetic pathways in the initiation and advancement of AML (2). These alterations in epigenetic regulation can lead to aberrant gene expression profiles, independent of alterations in the DNA sequence, thereby facilitating the pathogenesis of AML. Promising outcomes have been observed with the use of targeted inhibitors that modulate these epigenetic changes in AML treatment (37). The m6A modification stands out among the array of epigenetic alterations because of its prevalence as the most common RNA modification observed in mammalian mRNA (19). The addition of m6A to RNA is facilitated by the RNA methyltransferase complex comprising METTL3, METTL14, and WTAP, whereas its removal is performed by ALKBH5 or FTO. Acting as “reader” proteins for m6A, members of the YTH domain protein family regulate multiple RNA processes such as mRNA splicing, degradation, and translation. m6A RNA modification plays a crucial role in the development of hematopoietic cells, and its imbalance has been linked to the initiation of malignancies (38). In AML, the demethylases FTO and ALKBH5 are implicated in oncogenic functions (39, 40). Additionally, components of the methyltransferase complex, METTL3 and METTL14, have also been associated with AML progression (41–43). Furthermore, m6A reader proteins have been shown to regulate hematopoiesis and AML progression (44–47). In this study, we investigated the role and regulatory mechanisms of m6A methylation in ATOX1-mediated cuproptosis in AML. Through analysis using the RNA modification database RM2Target and subsequent experimental validation, we discovered that ALKBH5 could mediate m6A demethylation of *ATOX1*, promoting the degradation of *ATOX1* mRNA and thereby downregulating its expression. Additionally, overexpression of ALKBH5 was confirmed to inhibit cuproptosis in AML cells and suppress tumor growth by downregulating ATOX1 expression.

**Figure 8.**
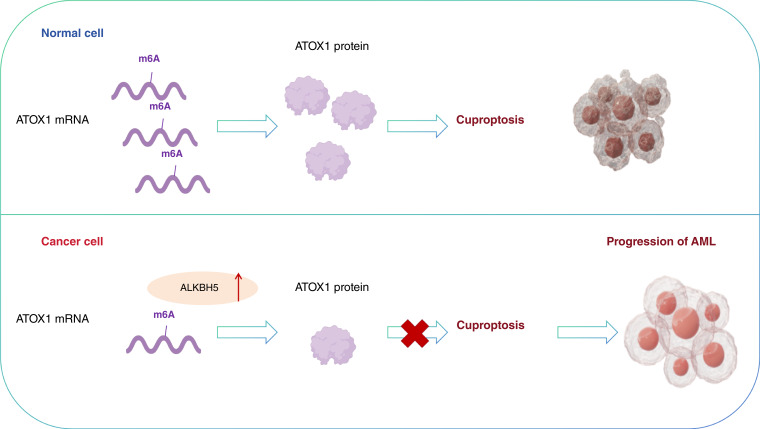
Schematic illustration of the proposed mechanisms: As depicted, ATOX1 undergoes m6A modification mediated by ALKBH5, which is upregulated in AML. This posttranscriptional modification destabilizes ATOX1 mRNA, leading to reduced ATOX1 expression. Consequently, the suppression of cuproptosis in AML cells is alleviated, thereby promoting AML progression.

In summary, our study revealed that ATOX1-mediated cuproptosis inhibits the growth of AML cells and, consequently, suppresses tumor progression. Furthermore, our findings have identified ALKBH5 as a mediator of m6A modification in *ATOX1*, thereby regulating its expression (Fig. 8). Our findings highlight the ALKBH5–ATOX1 axis as a mechanistically distinct but potentially targetable vulnerability in AML. Complementary to epitranscriptomic regulation of cell death, immunotherapy strategies such as nanobody-based CD70 chimeric antigen receptor T cells (48) exemplify the growing diversity of innovative treatments under development, underscoring the urgent need to integrate mechanistic and translational advances for improved AML therapy.

### Limitations in this study

Nevertheless, this study has several limitations. We employed shRNA transfection targeting ATOX1 for functional investigation, yet this approach did not fully deplete ATOX1 expression in cells. In future studies, we will utilize CRISPR/Cas9 to knock out ATOX1, thereby minimizing potential off-target effects. Additionally, although the subcutaneous xenograft model used here is effective for studying tumor cell–autonomous growth, it does not fully recapitulate the complexity of leukemia within the bone marrow microenvironment. Future research will employ more physiologically relevant models, such as tail vein injection or intra–bone marrow transplantation, to further validate the therapeutic potential of this pathway. Moreover, subsequent studies will directly measure intracellular copper levels via Inductively Coupled Plasma Mass Spectrometry and employ omics technologies such as RNA sequencing to comprehensively elucidate the transcriptional network regulated by ATOX1, with the aim of identifying novel therapeutic targets. Finally, investigating the role of the ALKBH5–ATOX1 axis in primary AML cells across different genetic subtypes will be an essential next step to confirm its broad clinical relevance.

## Supplementary Material

Figure S1 Figure S1. ATOX1 overexpression alleviates AML progression. AML cells were transfected with sh-ATOX1 or oe-ATOX1 for 7 days. A. CCK-8 assay for detecting the viability of AML cells. B. Flow cytometry for detecting the cell death of AML cells. Data are shown as the mean ± SD. n=3.

Figure S2 Figure S2. The rescue experiment verified the phenotypic specificity mediated by ATOX1 deletion. AML cells were transfected with sh-ATOX1 and/or oe-ALKBH5 with silent mutations. A. Western blot analysis of ATOX1 expression in AML cells. B. CCK-8 assay for detecting the viability of AML cells. C-D. EDU staining assay for detecting the cell proliferation of AML cells. Scale bar: 25 μm. E. Flow cytometry for detecting the cell death of AML cells. Data are shown as the mean ± SD. n=3.

Figure S3 Figure S3. ALKBH5-mediated m6A modification regulating ATOX1 expression affects cuproptosis in AML cells. A. CCK-8 assay for detecting the viability of AML cells transfected with oe-NC or oe-ATOX1 treated with different concentrations of DSF/Cu (0 nM, 2 nM, 10 nM, 50 nM, and 200 nM) for 72 h. AML cells transfected with oe-ATOX1 and/or oe-ALKBH5 were treated with 200 nM DSF/Cu for 72 h. B. CCK-8 assay for detecting the viability of AML cells. C. EDU staining assay for detecting the cell proliferation of AML cells. Scale bar: 25 μm. D. Flow cytometry for detecting the cell cycle of AML cells. E. Flow cytometry for detecting the cell death of AML cells. F. Western blot analysis of lipoylated DLAT expression in AML cells. G. Western blot analysis of Fe-S cluster proteins (FDX1 and LIAS) expression in AML cells. Data are shown as the mean ± SD. n=3.

## Data Availability

All data are available in the main article, supplemental files, or from the corresponding author on reasonable request.
