# Supplementary material for: m6A Modification of ATOX1 Inhibits Acute Myeloid Leukemia Progression by Promoting Cuproptosis
Source: Cancer Res Commun. 2026 Apr 1;6(4):714–27. doi: 10.1158/2767-9764.CRC-25-0436 (PMC13040171; doi:10.1158/2767-9764.CRC-25-0436)
Supplement: Figure S1 — ATOX1 overexpression alleviates AML progression. AML cells were transfected with sh-ATOX1 or oe-ATOX1 for 7 days. A. CCK-8 assay for detecting the viability of AML cells. B. Flow cytometry for detecting the cell death of AML cells. Data are shown as the mean ± SD. n=3. [file crc-25-0436_figure_s1_suppsf1.docx]

**
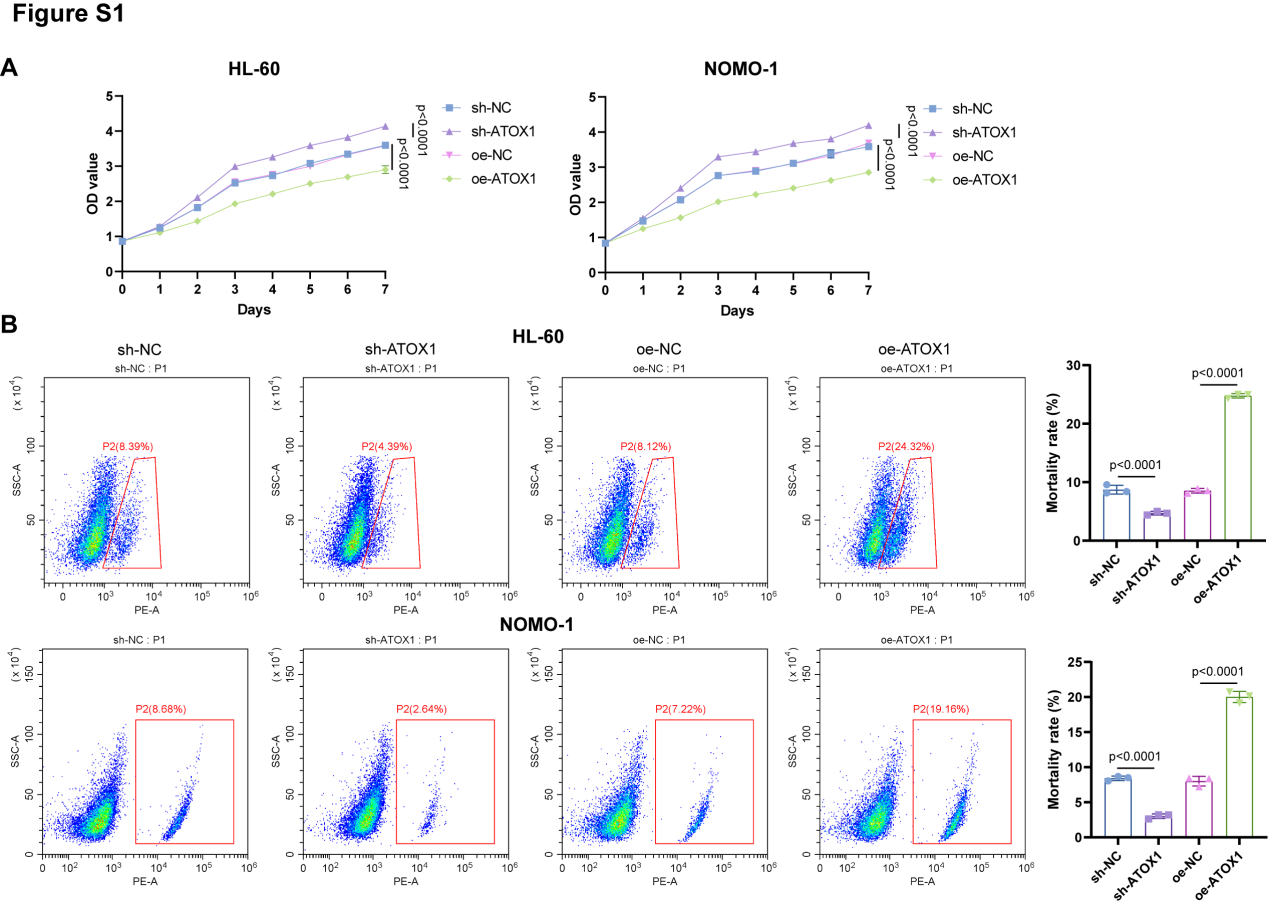
Figure S1. ATOX1 overexpression alleviates AML progression.** AML cells were transfected with sh-ATOX1 or oe-ATOX1 for 7 days. A. CCK-8 assay for detecting the viability of AML cells. B. Flow cytometry for detecting the cell death of AML cells. Data are shown as the mean ± SD. n=3.
