# Supplementary material for: m6A Modification of ATOX1 Inhibits Acute Myeloid Leukemia Progression by Promoting Cuproptosis
Source: Cancer Res Commun. 2026 Apr 1;6(4):714–27. doi: 10.1158/2767-9764.CRC-25-0436 (PMC13040171; doi:10.1158/2767-9764.CRC-25-0436)
Supplement: Figure S2 — The rescue experiment verified the phenotypic specificity mediated by ATOX1 deletion. AML cells were transfected with sh-ATOX1 and/or oe-ALKBH5 with silent mutations. A. Western blot analysis of ATOX1 expression in AML cells. B. CCK-8 assay for detecting the viability of AML cells. C-D. EDU staining assay for detecting the cell proliferation of AML cells. Scale bar: 25 μm. E. Flow cytometry for detecting the cell death of AML cells. Data are shown as the mean ± SD. n=3. [file crc-25-0436_figure_s2_suppsf2.docx]

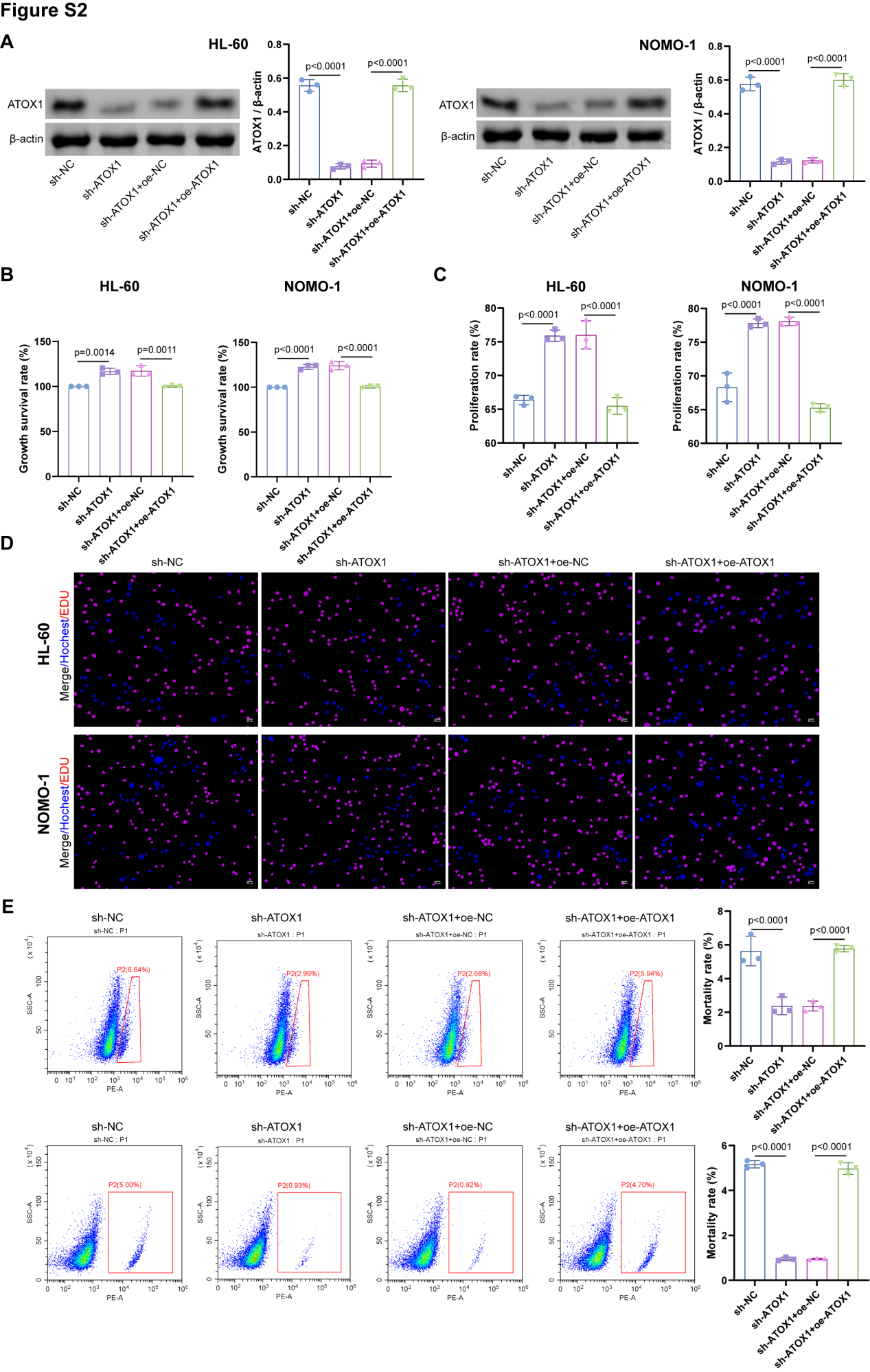


**Figure S2. The rescue experiment verified the phenotypic specificity mediated by ATOX1 deletion.** AML cells were transfected with sh-ATOX1 and/or oe-ALKBH5 with silent mutations. A. CCK-8 assay for detecting the viability of AML cells. C. EDU staining assay for detecting the cell proliferation of AML cells. Scale bar: 25 μm. D. Flow cytometry for detecting the cell death of AML cells. Data are shown as the mean ± SD. n=3.
