# Supplementary material for: m6A Modification of ATOX1 Inhibits Acute Myeloid Leukemia Progression by Promoting Cuproptosis
Source: Cancer Res Commun. 2026 Apr 1;6(4):714–27. doi: 10.1158/2767-9764.CRC-25-0436 (PMC13040171; doi:10.1158/2767-9764.CRC-25-0436)
Supplement: Figure S3 — ALKBH5-mediated m6A modification regulating ATOX1 expression affects cuproptosis in AML cells. A. CCK-8 assay for detecting the viability of AML cells transfected with oe-NC or oe-ATOX1 treated with different concentrations of DSF/Cu (0 nM, 2 nM, 10 nM, 50 nM, and 200 nM) for 72 h. AML cells transfected with oe-ATOX1 and/or oe-ALKBH5 were treated with 200 nM DSF/Cu for 72 h. B. CCK-8 assay for detecting the viability of AML cells. C. EDU staining assay for detecting the cell proliferation of AML cells. Scale bar: 25 μm. D. Flow cytometry for detecting the cell cycle of AML cells. E. Flow cytometry for detecting the cell death of AML cells. F. Western blot analysis of lipoylated DLAT expression in AML cells. G. Western blot analysis of Fe-S cluster proteins (FDX1 and LIAS) expression in AML cells. Data are shown as the mean ± SD. n=3. [file crc-25-0436_figure_s3_suppsf3.docx]

**
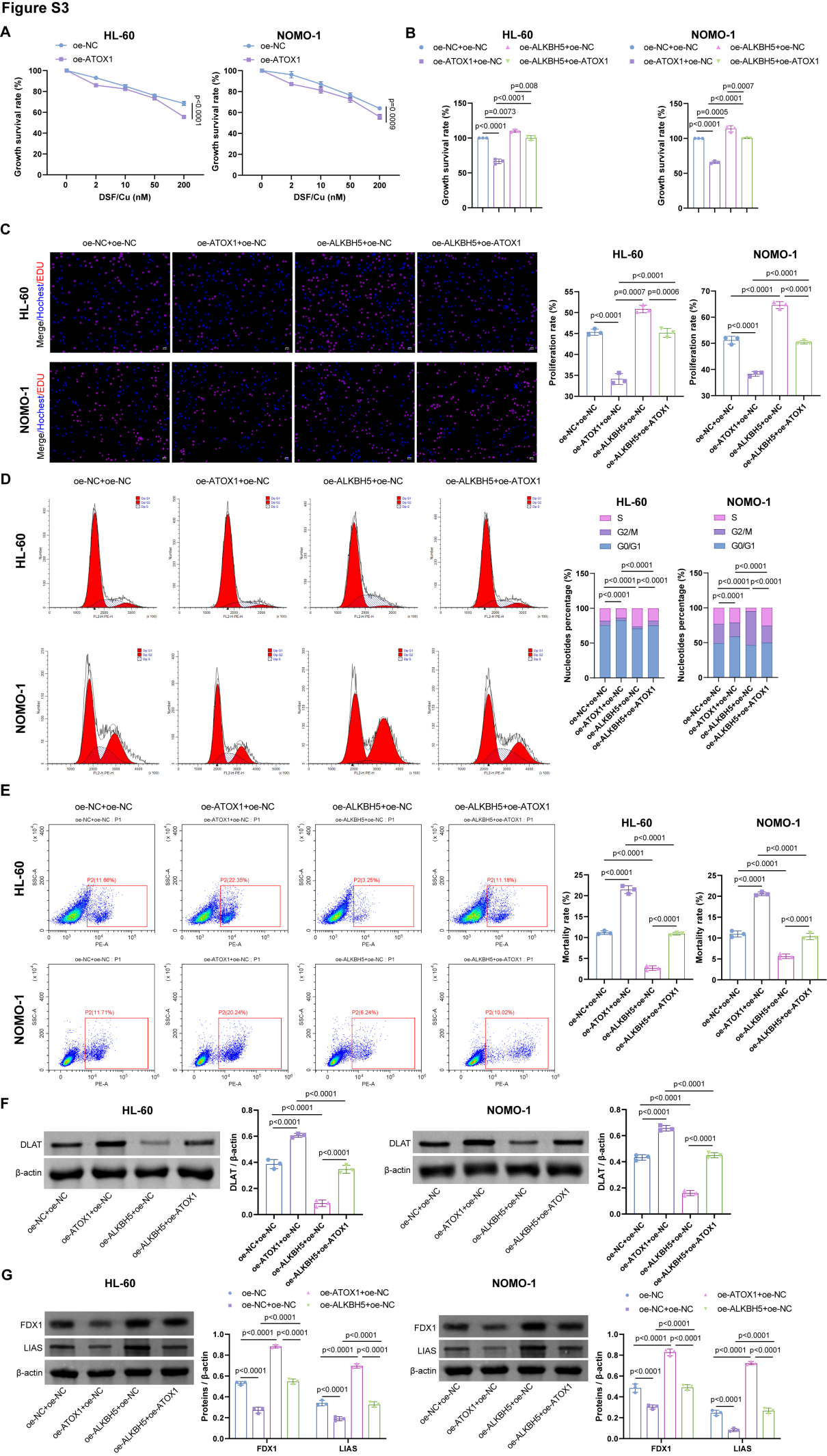
**

**Figure S3. ALKBH5-mediated m6A modification regulating ATOX1 expression affects cuproptosis in AML cells.** A. CCK-8 assay for detecting the viability of AML cells transfected with oe-NC or oe-ATOX1 treated with different concentrations of DSF/Cu (0 nM, 2 nM, 10 nM, 50 nM, and 200 nM) for 72 h. AML cells transfected with oe-ATOX1 and/or oe-ALKBH5 were treated with 200 nM DSF/Cu for 72 h. B. CCK-8 assay for detecting the viability of AML cells. C. EDU staining assay for detecting the cell proliferation of AML cells. Scale bar: 25 μm. D. Flow cytometry for detecting the cell cycle of AML cells. E. Flow cytometry for detecting the cell death of AML cells. F. Western blot analysis of lipoylated DLAT expression in AML cells. G. Western blot analysis of Fe-S cluster proteins (FDX1 and LIAS) expression in AML cells. Data are shown as the mean ± SD. n=3.
